# Local climate change cultures: climate-relevant discursive practices in three emerging economies

**DOI:** 10.1007/s10584-019-02477-8

**Published:** 2019-07-09

**Authors:** Nick Nash, Lorraine Whitmarsh, Stuart Capstick, Valdiney Gouveia, Rafaella de Carvalho Rodrigues Araújo, Monika dos Santos, Romeo Palakatsela, Yuebai Liu, Marie K. Harder, Xiao Wang

**Affiliations:** 1 School of Psychology, Cardiff University, Cardiff, UK; 2 Department of Psychology, Federal University of Paraiba, João Pessoa, Brazil; 3 School of English and Media Studies, Massey University, Wellington, New Zealand; 4 Department of Psychology, University of South Africa, Pretoria, South Africa; 5 RI Ethnographic Research Studio, London, UK; 6 Fudan Tyndall Center, Department of Environment and Engineering, Fudan University, Shanghai, China

## Abstract

In recent decades, greater acknowledgement has been given to climate change as a cultural phenomenon. This paper takes a cultural lens to the topic of climate change, in which climate-relevant understandings are grounded in wider cultural, political and material contexts. We approach climate-relevant accounts at the level of the everyday, understood as a theoretically problematic and politically contested space This is in contrast to simply being the backdrop to mundane, repetitive actions contributing to environmental degradation and the site of mitigative actions. Taking discourse as a form of practice in which fragments of cultural knowledge are drawn on to construct our environmental problems, we investigate citizens’ accounts of climate-relevant issues in three culturally diverse emerging economies: Brazil, South Africa and China. These settings are important because greenhouse gas (GHG) emissions are predicted to significantly increase in these countries in the future. We conducted semi-structured interviews with a range of citizens in each country using a narrative approach to contextualise climate-relevant issues as part of people’s lifestyle narratives. Participants overwhelmingly framed their accounts in the context of locally-salient issues, and few accounts explicitly referred to the phenomenon of climate change. Instead, elements of climate changes were conflated with other environmental issues and related to a wide range of cultural assumptions that influenced understandings and implied particular ways of responding to environmental problems. We conclude that climate change scholars should address locally relevant understandings and develop dialogues that can wider meanings that construct climate-relevant issues in vernacular ways at the local level.

**Electronic supplementary material:**

The online version of this article (10.1007/s10584-019-02477-8) contains supplementary material, which is available to authorized users.

## Introduction

Material impacts from climate change place huge stresses on the planet’s biophysical systems (Nolan et al. 2018), necessitating wide-ranging shifts towards the decarbonisation of societies in order to limit these impacts (Dubois et al. 2019). In addition, climate change is also a cultural phenomenon, whereby understandings of, and responses to climate change are interpreted in relation to wider sociocultural, political and material contexts (Baer and Singer 2018; Goldman et al. 2018; Geoghegan and Leyson 2012; Hulme 2016, 2008). Following Geoghegan and Leyson’s (2012) assertion that greater attention to the influence of culture is needed to understand individual and community responses to climate change, this paper takes a cultural lens to examining talk about climate change and other environmental issues in accounts from citizens in three culturally diverse settings. Cultural psychological approaches seek to understand how culture and identity influence individual cognition and behaviour in different ways (Markus and Kitayama 2003). In doing so, culture and mind are treated as mutually constitutive and to some degree, inseparable (Fiske et al. 1998; Shweder, 1991). We define culture primarily as constituted through discourse in which an attention to acts of communication can reveal aspects of culture through particular events, practices and ideas (Carbaugh 2007). We therefore view culture as fragmented, contested and constituted through discursive acts, as opposed to something that surrounds a society (Hulme 2016). From a cultural geographical perspective, constructions of knowledge, social relations and identities are also connected to material and spatial realities and their politics (Geoghegan and Leyson 2012; Brace and Geoghegan 2011). We begin by outlining two broad ways of defining climate and climate change, referred to here as the ‘scientific’ and ‘cultural’ perspectives, each with its own ontological and epistemological foundations. We then proceed by considering the slippery concept of culture, and discuss the context of ‘*everyday life*’. We argue that the everyday is not merely the backdrop to repeated mundane forms of practice, but as a space in which the cultural phenomenon of climate change both constitutes and is constituted by tacit everyday realities, identities and practices in cyclic relationship (Kothari and Arnall 2019). We then introduce an interview study of everyday accounts of climate change and other environmental issues voiced by citizens in Brazil, South Africa and China. In doing so, we illustrate how climate change can be constituted and understood through culturally embedded forms of discourse, in which communication constitutes a type of cultural practice.

### Scientific and cultural approaches to climate and climate change

Developments in post-structural and critical theory within the social sciences and humanities have challenged scientific theory regarding the production of knowledge and claims to objectivity and truth (Rice et al. 2018; Hulme 2008). Perspectives from cultural geography, sociology, anthropology and cultural psychology assert that the human experience is inextricably situated within wider social and political contexts, in which understandings of ‘natural’ phenomena are invariably ‘cultural’ (Salvatore 2018). Within social psychology, the cultural and critical turns have also led to a critique of the deterministic focus on internal psychological factors, the primacy of individual agency as underpinning social phenomena, and a reluctance to engage with wider socio-political debates (Batel et al. 2016). Consequently, cultural psychology has emerged as a psychological approach that looks beyond the individual, to external forces as shaping behaviour (Salvatore 2018). However, within the wider social sciences and humanities, culturally grounded approaches have gained more traction, more fully acknowledging the influence of sociocultural factors, temporalities and spatialities on systems of knowledge production, including pressing social issues such as climate change (Hulme 2016; Brace and Geoghegan 2011). In recent decades, climate change has undergone a process of diffusion from the natural sciences to the social sciences and humanities, in which climate change has shifted in meaning from a purely natural phenomenon to having a cultural dimension (Hulme 2016; Brace and Geoghegan 2011; Batterbury 2008). Scientific definitions of climate have emerged primarily from statistical abstractions and the development of climate models based on an accumulation of observational data over time (Hulme 2016, 2008), which has formed the foundation of national and international policy and governance (Brace and Geoghegan 2011). These are premised on a philosophical separation of nature and culture, as well as the assumption that climate and climate change can only be studied, explained and predicted through scientific theory and observation (Hulme 2016).

However, recent work in the social sciences and humanities has sought to challenge the dominant scientific approach in favour of one that recognises the cultural, historical and politically contested settings in which knowledge about climate is produced (Hulme 2016, 2015, 2008). This is not to privilege alternative forms of climate knowledge over science, but to recognise that climate may be comprehended in multifaceted ways (Leyshon 2014; Popke 2016; Yeh 2016). This alternative rendering of climate views knowledge as created within specific cultural contexts and emerging from alternative ways of knowing, such as culturally held knowledge and sensory experience of one’s surroundings (Hulme 2016). Such knowledge is in constant flux, in response to material and technological changes, the creation of novel ideas and new ways of relating socially (Hulme 2015). This entails a shift in the way we understand climate from a global physical systemic perspective, to one constructed within frameworks of localised forms of deep symbolic human meaning and sensory experience (Hulme 2015). Embracing methodological tensions and alternative forms of climate knowledge creates opportunities for building diverse communities of action and social transformations grounded within multiple forms of practice and knowledge (Rice et al. 2018).

A cultural perspective on climate views both culture and climate as mutually constitutive and cyclic, whereby natural phenomena both impacts on culture and is impacted upon by culture (Kothari and Arnall 2019). At a fundamental level, culture is constituted by ideas, practices and objects that create networks of shared meaning in time and space (Hulme 2016). Moreover, culture does not determine individuals, but provides tools that can be flexibly drawn on in constructing understandings of the world (Adger et al. 2012; Rudiak-Gould 2012). Culture is not something that exists out there in the world but is reproduced in fragments through human practices. Taking communication as a form of practice (Hepburn and Potter 2006), cultural meanings are an integral element of discourse; therefore, culture both shapes communication and is constituted by discursive practices, making communication a primary locus of culture (Carbaugh 2007). In communicating with others in daily life, speakers draw upon specific ideas, events and actions in which cultural meanings are emergent and contested. Such narratives used to communicate climate change understandings often take the form of stories, in which accounts are framed in ways that have rhetorical implications (Fløttum and Gjerstad 2017). Identifying cultural elements of discourse requires attention to the interactional accomplishments, structural features and sequential organisation of discursive accounts (Carbaugh 2007). While structuring understandings, experience and responses to phenomena such as climate and climate change, cultural ideas may nonetheless remain tacit and transparent to speakers and their interlocuters (Quinn 2016). Of course, cultures cannot be defined in line with national borders; in addition, multiple cultures may operate within a given place at different social and geographical scales (Hulme 2009). In approaching culture, we envision this in a fragmented sense, accessible by different groups.

### Climate understandings and everyday life

Though climate trends are more or less beyond the scope of the lay observer, climate change has permeated the spaces of the everyday in ways that are highly politicised and contested, and which support individual, collective and institutional responses (Brace and Geoghegan 2011). Such knowledge is culturally situated and relational; that is, climate change becomes significant for individuals in relation to other kinds of environmental and culturally salient issues rather than as an issue in itself (Clifford and Travis 2018; Boykoff et al. 2009; Macnaghten 2003). Consequently, a growing body of studies mainly from within cultural geography, anthropology and sociology, have sought to examine the climate-relevant understandings and responses of individuals and communities in different places (for example, Barnes and Dove 2015; Adger et al. 2012; Brace and Geoghegan 2011; Norgaard 2011). These situated, micro-approaches have sought to reveal alternative forms of knowing climate at the level of everyday life (O’Neill and Nicholson-Cole 2009).

The ‘*everyday*’ forms a key concept in cultural studies and is broadly defined as the site of tacit knowledge and ongoing mundane practices shared by ordinary people (Heller 2015; Eriksen et al. 2015; Sztompka 2008). Other scholars have sought to problematise the common notion of everyday life, upon which so much cultural analysis is based. Lippuner (2004) remarks on the difficulties of observing the everyday, commenting that it comprises an idealised construction for the purposes of observing culture on the ground, in which the everyday approximates to some kind of actual material reality. As such, it is constituted by its status as ‘other’ to the academic observer. Contrary to its repetitious, settled nature, Fiske (1992) notes the epistemic volatility and ephemerality of everyday life. Meanwhile, Kothari and Arnall (2019) suggest that as a hyper-cultural space, the everyday implicitly brackets out nature and marginalises the non-human. In addition, the everyday is preoccupied with the here-and-now, rather than being future-oriented in ways commensurate with environmental issues (cf. Maniates 2012).

While the everyday remains a contested space, scholars have remarked on the importance of the everyday as a site where cultural ideas and practices are normalised and remain unquestioned (Bourdieu 1977). Everyday practices can conceal patterns of inequality and oppression that are taken for granted (De Certeau 1984). MacGregor (2014) discusses how dominant neoliberal forms of everyday climate-relevant practice obscure alternative forms of lived experience and understandings that diverge from constructions of the ideal neoliberal citizen-consumer. Other work has also shown how climate-relevant relationships between knowledge, practice and everyday spaces strategically attribute responsibility for addressing environmental problems (Bee et al. 2015). It is therefore through the lens of everyday life that the politics and power relations of culture are exposed (Gram-Hanssen 2011). Conflicts between expert and more colloquial perspectives underline the importance of considering the filtering effects of local context and experience when communicating about climate change (Wolf and Moser 2011).

While weather and climate remain categorically different phenomena, sensory experience of weather is a primary way in which people come to know climate (Hulme 2016; Leyshon 2014; Knebusch 2008; Adger et al. 2009). Extreme weather events, seasonal changes and related changes in the physical landscape, flora and fauna indicate climate changes for lay observers, though weather is always in flux (Hulme 2016). Studies have documented accuracy in lay observations of precipitation over time (Chaudhary et al. 2011), as well as significant levels of disagreement in observations of weather between different observers (Byg and Salick 2009), and inaccuracies in lay observations of weather (Manandhar et al. 2011). Experience of weather and its material and psychological effects condition individual and collective responses and adjustments to atmosphere and climate in culturally and spatially situated ways that become relevant and routinised in the everyday (de Vet 2014; Ingold 2007). People may also know climate in other ways, including the media (Boykoff et al. 2009; Boykoff and Boykoff 2007), formal education (Muttarak and Lutz 2014), climate politics (Whitmarsh and Capstick 2018) and through everyday practices. For example, daily mobility decisions (for example, transport modes, travel times and routes) are affected by meteorological parameters, thereby facilitating and constraining daily routines and influencing climate understandings (Kothari and Arnall 2019).

In summary, we approach everyday understandings of climate-relevant issues as cultural phenomena, whereby culture is instantiated in communication as a form of cultural practice. In accordance with a cyclic, mutually constitutive approach to climate as a cultural phenomenon, these cannot be separated and can be observed in accounts of material conditions and situated life experiences such as commuting, work and leisure, that both determine those practices and concomitantly shape climate understandings on the basis of that locally grounded, culturally rich experience (Clifford and Travis 2018; Brace and Geoghegan 2011; Geoghegan and Leyson 2012; Rudiak-Gould 2012).

We investigate accounts of everyday climate relevance in three culturally diverse emerging economies: Brazil, South Africa and China. These were chosen because carbon emissions are set to rise significantly in these countries as a consequence of accelerating economic development (Hallding et al. 2013; Rong 2010). Wider cultural change, for example, through economic development and industrialisation, can lead to lifestyles becoming more energy- and resource-intensive (Wolf and Moser 2011). Encouraging modest, voluntary lifestyle change could considerably reduce a nation’s emissions (Dietz et al. 2009). In addition, each country faces significant climate change risks and other environmental issues; in South Africa, climate change is expected to lead to higher temperatures and drier landscapes, along with more frequent extreme weather events, including heatwaves and drought (Ziervogel et al. 2014). Brazil faces unprecedented deforestation and species loss in the Amazon Basin, as a consequence of government policy to convert natural ecosystems to agricultural land, and intensification of land use (Lambin et al. 2003). The north-east of the country is perennially subject to low rainfall and drought, and projected to increase in frequency and severity in the future (Bedran-Martins et al. 2017). Meanwhile, the scale and rapidity of industrial development within China has created an array of environmental and climate change–related risks, including land degradation (Zhang et al. 2007), and food and water security (Lu et al. 2015).

Insights into everyday understandings of climate change and environmental issues in economically transitioning cultures can yield valuable insights on factors that might facilitate or obstruct climate change engagement. For example, a lack of response to climate change may be due to wider cultural issues linked to wider issues of culture, identity and lifestyle rather than a lack of concern (Norgaard 2011; Howell 2013). The environmental sustainability of a society requires the coordination of a society at multiple levels, including its citizens (Adger et al. 2012). Emerging economies represent important, but under-researched economic and cultural contexts in relation to climate change (Hallding et al. 2013). Greenhouse gas (GHG) emissions in existing industrialised economies currently account for less than 50% of the global total and are projected to decrease in future. Conversely, emissions are predicted to substantially increase in emerging economies (Hallding et al. 2013). This also follows Article 12 of the Paris Agreement, which highlights the importance for governments to generate the capacity for citizens to engage with climate change and support wider action in the course of their daily lives.

While limiting generalisability across contexts, such approaches generate richer and more contextually detailed accounts of environmental engagement (Hargreaves 2011), as befitting cultural perspectives. We also attempt to avoid the methodological individualism of other psychological approaches that neglect the wider sociocultural contexts that constrain climate-relevant understandings (Batel et al. 2016). Following other approaches that take a middle position between ‘behaviour’ and ‘practices’ (for example, Thomas et al. 2017), we retain a psychological focus on the individual and behaviour, though with a recognition this is negotiated through culturally specific and embodied understandings, analogous to the materials, meanings, and competences forming practices (Shove et al. 2012). Cultural accounts also require an appreciation of the active, functional ways in which people construct and interpret climate change and their worlds more generally. There is no straightforward window to describe reality as it is, whether through the framework of behaviours or practices. Accounts are inevitably subjective and positioned, representative of social performance rather than direct representations of the world as it is (Potter 1996). Psychological accounts are less expressions of internalised mental states than culturally available ways of understanding the world (Billig 1996).

Our approach is restricted to an analysis of climate-relevant issues as raised by our participants, which balances more formalised scientific concepts of climate change against a recognition that, for non-experts, this topic area is not neatly delineated and may span multiple concerns. We therefore acknowledge the ‘unavoidable trade-offs’ (Rudiak-Gould 2012: 46), that occur in representations of non-expert perspectives on climate change. Furthermore, our analytic materials were derived from a broader programme of work in which the focus was on environmentally sustainable lifestyles in the round. In this paper, we have identified cases deemed to be climate-relevant, either directly (for example, perceptions of changing weather conditions) or indirectly (for example, concerns about localised air pollution and perceived responsibility for addressing this). Based on our review of the literature, our research questions are as follows:

- How do citizens in Brazil, South Africa and China understand climate change and other environmental issues in the contexts of their everyday lives?
- How are climate-relevant and environmental issues constructed in discourse in relation to wider cultural ideas with relevance to negotiating these issues?
- How do such accounts position speakers with reference to negotiating climate change and other environmental issues in daily life?

## Method

The following sections summarise the fieldwork design and procedure, which was approved by Cardiff University’s School of Psychology Ethics Committee. Fieldwork comprised semi-structured qualitative interviews with a range of citizens in each of the three countries studied. Interviews were designed broadly to elicit discussions regarding perceptions of and responses to environmental issues, including climate change.

### Participants

Interviews in the three countries were conducted at periodic intervals between March 2015 and April 2016. A purposive sampling strategy was created to ensure that we spoke with a range of sociodemographically diverse participants in each country as was possible. All participants were aged 18+. To generate a range of environmental perspectives in each country, we recruited a subsample of citizens who were less environmentally engaged and a subsample who were more environmentally engaged. In recruiting the former subsample, we worked with academic collaborators in local institutions who advertised the study locally as a ‘behaviour and lifestyle perceptions’ study, avoiding any mention of environmental issues and using some simple screening questions. To recruit the latter subsample, we approached local environmental organisations and asked whether we could interview employees who were environmentally engaged. Table 1 shows basic demographic details for each group.

**Table 1:** Participant demographic details

		Less environmentally engaged		More environmentally engaged		All	
Brazil							
Gender	Female	10	58.8%	9	50%	19	54.3%
	Male	7	41.2%	9	50%	16	45.7%
Age group	18–24	4	23.5%	0	0%	4	11.4%
	25–34	3	17.6%	5	27.8%	8	22.9%
	35–44	2	11.8%	10	55.6%	12	34.3%
	45–54	4	23.5%	3	16.7%	7	20%
	55–64	0	0%	0	0%	0	0%
	65+	4	23.5%	0	0%	4	11.4%
China							
Gender	Female	9	64.3%	12	70.6%	10	32.3%
	Male	5	35.7%	5	29.4%	21	67.7%
Age group	18–24	4	28.6%	4	23.5%	8	25.8%
	25–34	7	50%	8	47.1%	15	48.4%
	35–44	2	14.3%	0%	0%	2	6.5%
	45–54	1	7.1%	3	17.6%	4	12.9%
	55–64	0	0%	0	0%	0	0%
	65+	0	0%	2	11.8%	2	6.5%
South Africa							
Gender	Female	8	47.1%	10	62.5%	18	54.5%
	Male	9	52.9%	6	37.5%	15	45.5%
Age group	18–24	1	5.9%	1	6.3%	2	6.1%
	25–34	13	76.5%	5	31.3%	18	54.5%
	35–44	2	11.8%	7	43.8%	9	27.3%
	45–54	1	5.9%	1	6.3%	2	6.1%
	55–64	0	0%	0	0%	0	0%
	65+	0%	0%	2	12.5%	2	6.1%

Fieldwork in Brazil took place during March/April 2015 in two separate locations (*n* = 35). Interviews with less engaged participants were conducted in the coastal city of João Pessoa, Paraiba, in north-east Brazil (population 720,000). This group comprised local residents recruited by our collaborators at the Federal University of Paraiba (*n* = 17). Participants were screened to ensure that they were aged 18+, did not have any significant pro-environmental interests or commitments and did not work in the environmental sector. We also purposively selected participants to ensure we had a roughly 50% gender split and some variation in terms of age.

Interviews with more engaged participants took place in the country capital, Brasilia (population 2.481 million), and were recruited by local collaborators at the World Wildlife Fund for Nature (WWF) Brasilia Office (*n* = 18). An advert for the study was circulated to all employees, which explicitly mentioned our interest in speaking with environmentally committed individuals (as some members of staff were employed by WWF in non-environmental capacities). We also used a couple of simple screening questions to ensure this, as well as trying to maintain a 50% gender split and variation in age.

Fieldwork in China took place during March 2016 in the city of Shanghai on the eastern coast (population 24.18 million). As we were unable to secure the collaboration of an environmental organisation, both groups were recruited by two collaborators, a freelance ethnographer based in Shanghai and collaborators at Fudan University. The less engaged group was recruited through an online advert posted on local social media networks, following the same screening procedure as above (*n* = 15). The more engaged group was recruited through an online advert on the ‘Shanghai Green Initiatives’ network on the WeChat social media app (*n* = 15). A range of environmentally committed citizens responded, principally environmental sector employees, volunteers and consultants.

Fieldwork in South Africa took place during September 2015 in two separate locations (*n* = 34). Less engaged participants were recruited in the city of Pretoria (population 742,000) by our collaborators at the University of South Africa (UNISA), using the same purposive sampling and screening approach as in João Pessoa (*n* = 17). For the more engaged group, we again collaborated with WWF, this time in their Cape Town Office. Participants were recruited using an internally circulated advert for the study and then screened as described above in the fieldwork with WWF Brasilia (*N* = 17).

### Fieldwork procedure

Following recruitment, citizens were invited to participate in an interview lasting up to 1.5 hours to discuss specific issues relating to their everyday lives; while we disclosed our interest in environmental issues in relation to people’s lives to more engaged participants, we did not explicitly mention environmental issues to less engaged participants prior to interview.

In Brazil and South Africa, interviews with less engaged participants took place in interview rooms at the collaborating academic institutions and interviews with more engaged participants were conducted in private offices at WWF. In China, the majority of interviews took place in a rented meeting room in central Shanghai or at Fudan University. However, due to the size of the city, some participants were unable to travel to this location or the collaborating institution and so we held interviews in other locations. For the less engaged group, two interviews took place elsewhere (one in a quiet café, the other in a participant’s home). For the more engaged group, six interviews were conducted in private and secluded spaces in participant workplaces.

Before each interview began, we obtained written informed consent from all participants. The interview team comprised a male researcher in South Africa, where interviews were conducted in English, and the same male researcher and different female translators in Brazil and China where interviews were conducted in the local language unless participants elected to speak in English. Translators took an active part in the interviews, checking participant understanding, clarifying questions and responses and elaborating on cultural nuances, as opposed to simply translating questions and answers.

A semi-structured interview approach was used (King and Hugh-Jones 2018), in which a standard question protocol was applied in each country, which also allowed for follow-up questions and orientation to topics of interest raised by participants (see Supplementary Material). This flexibility made the semi-structured method particularly suited to multisited, cross-cultural fieldwork (Hagaman and Wutich 2017), while also allowing for the generation of contextual detail (McIntosh and Morse 2015). The interview approach was also informed by the episodic narrative method (Flick 2000), which focuses on eliciting descriptions of anecdotes and episodes from people’s lives as ways of making sense of the world. An ‘in-interview’ method of translation was employed in which questions and responses were translated to and from English by the translator (except where participants opted to speak in English).

Interviews were audio-recorded and subsequently translated into English where appropriate and transcribed. The interviewer also made written field notes. We used template analysis (Brooks et al. 2015) to identify and code themes in participants’ accounts. Of particular interest were utterances referring to issues of relevance to the intersection of climate-relevant issues and everyday lifestyles. The interview audio and texts were analysed using NVivo 11, supplemented by the written field notes. In the “Analysis” section, quotes are labelled ‘*Direct*’ if spoken in English, or ‘*Transl.*’ if translated from another language (either by the translator in the interview or during transcription).

## Analysis

The interview extracts that appear below illustrate features of interest that may be applicable across more than one country. In such instances, we present a single extract and allude to its occurrence in other cultural contexts in the text. We now move on to discuss these accounts in more detail.

### Experiencing climate change

In furnishing accounts of environmental issues in the context of their day-to-day lives, participants in all three countries rarely oriented explicitly to the topic of climate change spontaneously. This follows Rudiak-Gould’s (2012) observation of the often-messy delineation of non-expert accounts of climate change. Environmental conditions analogous to climate change were mentioned though not categorised as such. In these descriptions of environmental conditions, the majority of participants referred to the local environment. Following Geoghegan and Leyson (2012), weather and the physical landscape served as primary indicators of the state of the local environment. In the following account from South Africa, the speaker does not mention weather, but describes how features of the local landscape had degenerated over time due to climatic change:

Participant [Direct]: ‘I know one place, Kumala … apparently the climate changed, you know it was just a nice environment, apparently it was okay, then the climate changed, now apparently they saying the soil it’s kind of like black, and it is always dusty there … they even told people that they’re not allowed to stay there like no people are allowed to stay, it’s harmful to people and all those things, due to the changing of the climate or something.’ (A12 less engaged group; Pretoria).

Adger et al. (2012) discuss cultural differences affecting the adaptive capacities of communities and the severity of climate change is conveyed through the enforced removal of the local community due to deteriorating conditions that had become harmful and were beyond the community’s adaptive capacity. In the next example from Brazil, the speaker’s account takes a national perspective but does not attribute environmental conditions to climate change or global warming; instead, a number of different environmental and cultural issues are combined in attributing cause for water shortages in the country:

Participant [Transl.]: ‘ … the things that he thinks that affect most the problem of water is that people tend to cut off a lot of trees illegally, and so because of that we haven’t had a lot of rain here in Brazil, and so because of that a lot of rivers are below their usual level or even higher, depending on the states. The few water that remains, people are polluting.’ (A10 less engaged group; João Pessoa).

The speaker discusses water as an environmental problem in Brazil, which is linked to a combination of cultural and natural antecedents, resulting in erratic rainfall and fluctuating river levels. This type of compounded environmental issue bears relation to Macnaghten’s (2003) assertion that personal experience of environmental issues is not simply experienced as a set of physical issues but entangled in cultural ideas and practices. Climate-relevant issues are also conflated with other significant environmental issues, such as illegal logging and water polluting practices—suggesting that this may set people up to believe in the wrong (or irrelevant) solutions, or to feel disempowered (for example, if environmental problems are caused by the actions of distant others, then there is nothing that can be done locally except to cope (Wolf and Moser 2011).

### Living with environmental risk

Perceptions of environmental problems were not constructed in terms of the issues themselves, but in terms of the spatial contexts associated with those problems. Bickerstaff and Walker (2003) discuss public constructions of air pollution in which air pollution is legitimated in certain contexts. In the following example from China, it was felt that living in a city was inevitably associated with some level of pollution to be expected and to which citizens were implicitly required to adapt:

Participant [Transl.]: ‘So there are always some reports about the air pollution, and over the news about the food safety crisis … I always think that if you live in a big city the air pollution is not avoided—it cannot be avoided. The bigger the city is, the serious the air pollution will be.’ (A1 less engaged group; Shanghai).

Such accounts are important as they invoke cultural assumptions about everyday spaces in ways that construct environmental issues as normal. Normalising environmental problems such as air pollution carries the risk that urban climate change and related impacts are likely to intensify as more people migrate to urban centres (Harlan and Ruddell 2011). The account is also constructed as a trade-off between environmental pollution and urban lifestyles—previous research in similar contexts has recorded citizens’ unwillingness to take personal responsibility for mitigation (Dong and Zeng 2018).

There was also evidence that despite the severity of some environmental risks, at the local level, it was the more conspicuous issues that were viewed as being most important. This is illustrated within the next extract, also from China, in which the problem of air pollution is acknowledged but contrasted favourably with other places, where air pollution is more serious. Conversely, it is the cleanliness of Shanghai’s streets that is more noticeable and objectionable:

Participant [Transl.]: ‘I think Shanghai in general is okay comparing to Beijing, which has a serious smog problem. The air pollution situation in Shanghai is okay, acceptable, but I think there are other issues when we take notice. For example, the streets in Shanghai is not that clean. So I think it’s a little bit dirty. I think it’s because the education level of the people living here is not sufficient high enough to change the behaviour. I think people kind of ignore the issue or something.’ (A7 less engaged group; Shanghai).

The relative importance of environmental problems was also formulated by participants in other ways. While climate change was recognised as one of the most significant problems facing society, the importance of the local context meant that other issues could be viewed as more important. In the following account, a participant in Brazil explains that while climate change was more important for people living in the Amazon, water shortages were more critical for people in large cities such as São Paulo. Moreover, these perceptions of environmental conditions guided behavioural responses:

Participant [Direct]: ‘I think it’s changing really fast. For example, everything today is related to climate change, carbon issues. In Brazil it’s becoming very critical, the issue of water security. That’s important because if I still lived in the Amazon I would be much more worried about carbon … but the scarcity of water in the Amazon is not an issue. Still we have to be responsible but it’s not the main issue. The main issue maybe is climate change. If you live in São Paulo probably water security becomes the big issue. So you have to - everybody would have to find - try to find the best solution to reuse, to not waste and all that. We do that a lot in Brasília too. So I think the issues depend a bit about the context, where you are, or the short-term problems, but I would say that these two issues are critical today in Brazil in general.’ (B11 engaged group; Brasilia).

Such examples are significant as they suggest that climate change may only resonate with citizens if they accord with locally significant problems. While wider issues were occasionally acknowledged, citizens were typically oriented towards issues of local rather than global concern. This contrasts with previous research (for example, Uzzell 2000), which measured greater concern for global issues across a range of cultures.

### Making a difference in daily life

While there were few notable differences between more and less environmentally engaged citizens, those who were more environmentally engaged tended to feel that there was little that they could do to make a difference to the environment through actions in their own lives, even at the local level. Negotiating environmentally responsible lifestyles was conveyed as being emotionally draining at times, with little hope in trying to bring about change as an individual. In the next extract, an environmentally engaged participant in Brasilia discusses her sense of futility, fuelled by living in a country in which environmental issues are a low priority for government and for the population more generally:

Researcher: ‘Do you feel like there’s anything you can personally do for the environment?

Participant [Direct]: No. I get very upset because I don’t think I can. Because even the people that work for it have a hard time. Me, myself, I will do it in a limited space at my house. But I don’t know how much that will be - it will be replicated, the little that we know, the little that we do among my kids. But that still won’t be enough to save the planet, let’s say … I am very worried, very concerned, yeah. Because little money or little attention is - especially in the country that, let’s say – let’s generalise it. Let’s say everybody, but in Brazil especially, at this moment not even education or health is being given attention. So the environment itself … I can teach maybe them how to survive, but I won’t save the planet.’ (B5 more engaged group; Brasilia).

Such accounts constructed everyday spaces as spaces of constraint, in which speakers were positioned as lacking the capacity to effect change due to a perceived lack of consensus among the wider public and governing institutions. For those who were more environmentally committed, there was a frustration with the lack of ecological citizenship shown by the majority of the population.

In the next extract from Brazil, the speaker talks about the ephemerality of felt responsibility for most citizens, manifested only at election time. Conversely, environmental issues were everyday issues requiring consistent attention and action on the part of the public; though citizens failed to make the connection between their lifestyle practices and their damaging impacts day in and day out:

Researcher: ‘What about as a citizen? Do you feel responsible to do environmental things in your personal life?

Participant [Direct]: Yes. Of course. Not only during elections. Because in Brazil we have this strange phenomenon. Most of the people feel this responsibility, this citizen’s responsibility only during the elections. After the elections the people used to, oh okay, I did my role, voting, okay. It’s really hard for us working with environmental questions, this kind of behaviour. Because we need citizenship during all the year, every day, every time. In Brazil it’s really hard to mobilise - is right to say?

Researcher: Yeah.

Participant [Direct]: … mobilise people to act on questions, on environmental questions. Because the people don’t used to see the direct links between them - day by day’. (B9 more engaged group; Brasilia).

Conversely, for less committed citizens, there was often a felt sense that the kinds of behaviours they currently did in everyday life were sufficient to address the environmental problems currently being faced. Notable is a disparity between the belief that the speaker can make a difference and concerns about the future of the planet, as the following example from South Africa illustrates:

Participant [Direct]: ‘Yeah I think the things that I do, they can make an impact because if you think about it, a lot of environmental problems that we are currently sitting with are caused by individual people, even if it’s companies or people with other vested interests, but they are caused by human negligence or human error, or whatever you might want to call it, but it’s individuals so having said that I think adhering to environmentally friendly behaviour can also bring about the opposite effect. If every individual takes it upon themselves to sort of like try and be environmentally more aware or conscious then that could have potential for good deeds towards the environment, because currently we’re sitting with a crisis and the rate that we are polluting the country or the world, we might not have a world to live in for our children’s children might not have a world to inherit, or they might inherit something which is completely dysfunctional and beyond repair, so I think yes it does in a way have an impact, what I’m doing.’ (A1 less engaged group; Pretoria).

These examples highlight the stark difference in terms of self-efficacy expressed by more and less engaged citizens. Though contradictory, each positions citizens as responsible for global scale environmental problems that threaten the planet’s future. This relates to an ongoing debate over the degree to which individuals, corporations and political institutions should bear responsibility for large-scale environmental problems (Batel et al. 2016).

### Lifestyles of frugality and environmental benefits

While participants did not demonstrate a high awareness of climate change, some were persuaded to engage in climate-relevant action motivated by non-environmental ideologies. Motivations were framed less in present circumstances and based more in the past, around narratives of formative childhood experiences. Being taught about the need to conserve scarce natural resources at an early age made a deep impression for some and instilled values that remained fixed in adulthood:

Participant [Direct]: ‘ … well I was raised by a father that was very conscious of environmental let’s say values, environmental impact and he always told us that we shouldn’t put lights on if it’s not necessary, we should save power, save water … so um that’s the type of environment I was raised in.’ (A3 less engaged group; Pretoria).

Accounts of everyday environmental engagement in the present sometimes drew on historical conceptions of livelihoods. Previous research has documented the salience of early childhood exposure to environmental messages to engagement in pro-environmental practices in adulthood (Chawla 1999). The accounts so far show that energy and natural resource conservation was embedded in narratives relating to citizens’ relationships and responsibilities to the state, and familial responsibilities, as opposed to a more straightforward concern about environmental conditions. Therefore, climate-relevant perceptions appear to depend on the fabric of everyday life as opposed to rational responses to an abstract physical phenomenon.

Climate-relevant narratives were also framed by ongoing national energy shortages, which had led to a rolling blackout throughout the country for several hours per day. While popular media representations of climate change had initially raised awareness about climate change, the experience of load-shedding had reprised a focus on the importance of energy conservation:

Participant [Direct]: ‘Al Gore’s documentary, yeah, um that sort of opened my eyes, you knew about global warming and you knew about all these things but it was out there you know, um so that made quite an impact on me, I remember watching that and thinking wow okay, and then there’s also things like strangely I guess the energy crisis here in South Africa at the moment also makes you think about conserving energy.’ (A2 less engaged group; Pretoria).

Understandings of wider cultural conditions were key to influencing motivations to engage in climate-relevant actions such as energy-saving, on the basis that engagement was as much in the national interest as the environmental interest. Previous work indicates that responses to energy shortages can be both positive and negative (Ngoma et al. 2018); however, examples such as the above indicate that energy shortages have a positive influence, at least in the short term.

### Experiencing environmental practices in different cultures

While the ideas and practices bound up in everyday life were taken for granted and transparent for citizens, a given cultural context, shifting between one context and another, could change perspective on tacit environmental ideas and practices, leading to consequent behaviour change. For example, travelling to another country in which environmental issues were given greater priority in everyday practices could sometimes lead citizens to change their ideas and behaviour when returning to their native culture. In the following extract from Brazil, the participant explains how travel alerted to her the lack of importance given by people in daily life back home which had led her to become more environmentally responsible:

Participant [Transl.]: ‘She thinks these are very important issues. She sees around this town, João Pessoa, that people do not see so much importance in these issues and they should. Travelling, she could see how people in other countries and other - in other places are worried about these issues. For example, trying to divide the garbage into wet garbage or dry garbage. So because of these travels she could have another vision about these issues and then she could develop a conscience about the environmental issues.’ (A15 less engaged group; João Pessoa).

This example is important as it illustrates, in a fairly direct way, how different cultural contexts mediate contrasting ideas about pro-environmental practices in daily life and how tasks such as dealing with domestic waste are enacted.

### Negotiating environmental knowledge in everyday life

Part of negotiating everyday life involved making decisions not only based on sensory experience of environmental conditions but on less visible, alternative sources of information. This was especially relevant in large cities where environmental conditions could not be accurately gauged through their visibility alone. In Shanghai in particular, while decisions about whether it was safe to go out on smoggy days relied significantly on official government forecasts of air quality, there was some doubt over the accuracy of the data used. According to some participants, official forecasts of atmospheric particulate levels were often manipulated in accordance with air quality targets to avoid sanctions. This meant that it was difficult for citizens to judge environmental conditions accurately, which had consequences for people’s everyday lives:

Participant [Direct.]: ‘Like people, they care about the environment, they really care about their environment, or they are well informed about the environmental index around them. Because in China some – well from my experience I can say the many indexes, they are not reliable. Like the water quality data, and yeah, air pollution, yeah … I don’t know the exact story, but maybe it’s tradition, like the Chinese people, they don’t value data that much as Western people … I mean the authentic data … I mean maybe they just feel it’s quite okay if they can do some manipulation of data … Because they need to report. They need to accomplish some – their own KPI (key performance indicators) … Yeah. If the data doesn’t meet some requirement, then they will get punished.’ (B12 more engaged group; Shanghai).

The above account indicates the difficulties of negotiating environmental problems in relation to the provision of environmental indicators, which are distrusted and skewed in order to meet officially sanctioned targets This is constructed as a cultural norm in China, whereby citizens do not trust data as in other cultures. In addition to negotiating environmental problems, an additional layer of complexity is therefore imposed in terms of building trust in official environmental information, the source of the information and the political context within which the information is situated.

## Conclusion

The preceding analysis outlines citizens’ accounts of negotiating climate-relevant issues in the contexts of their everyday lives. Talk about climate change was typically tied to other salient environmental and cultural factors, in line with previous work (for example, Rudiak-Gould 2012). This contrasts scientific understandings of climate change in which objective observations of climate are constructed in ways that lead to rational responses on the part of the wider society. Taking the perspective of culture as a form of discursive practice, participants’ accounts were not only about the phenomenon of climate change itself but were highly localised and relational—tied intimately to a range of locally significant issues. While global climate change and its impacts were occasionally acknowledged, climate change only really attained significance for people in relation to issues of concern more locally. Previous research has focused on perceptions of climate change based on observed changes in weather and seasons (for example, Geoghegan and Leyson, 2012), while our analysis suggests that understandings of climate change are based on an even wider range of issues of salience to citizens. In this paper, we have opened up novel connectivities between climate change and other spaces of the everyday. In situating accounts of climate-relevant issues in the context of the everyday and the spatial, participants were able to draw attention to other issues of significance in relation to their situated experience in which livelihoods and ways of living were being negotiated alongside climate change and other environmental problems. Moreover, these locally contingent aspects of climate change understandings were not only framed by broader issues in the here-and-now. Climate understandings were also tied to recollections of the past (for example, being raised in a background of poverty) and imagined futures (for example, feeling concerned about environmental collapse and eschatological fears).

With reference to speaker identities bound up in such accounts, narratives typically intoned themes of disempowerment and lack of capacity in terms of responding to climate-relevant issues, with reference to both uncontrollable forces of nature and social and political forces. Wider tacit assumptions positioned speakers in ways that constrained responses to environmental problems, underlining the everyday as a politically contested space reproduced through discursive practice and not simply the backdrop to mundane, repetitive practices (Kothari and Arnall 2019). This opens up the potential for the reconfiguration of problematic climate-relevant communicative practices and their cultural referents, going beyond the raising of awareness of climate change, which look likely to secure only marginal gains in shifting existing patterns of everyday life in more environmentally sustainable directions. This paper highlights some of the potential pitfalls in charging individuals with the responsibility for mitigating and adapting to climate change and instead calls for a deeper interrogation of the webs of meanings that structure environmental perspectives at the level of the everyday. At this early stage, our analysis underlines the importance of local cultural understandings and might at the very least foster ongoing dialogues that challenge the dominant cultural politics of climate change.

In a broader sense, emergent economies might also shape the everyday understandings of their citizens in a number of ways. For example, negotiating everyday life was associated with an acceptance of a certain level of environmental risk (for example, air pollution) as part of everyday life. One of the consequences of economic development is increasing urbanisation, which leads to more energy-intensive lifestyles (Sadorsky 2014). While economic development has important benefits for citizens, adaptation to certain levels of climate-relevant risk implies a potential trade-off whereby governments can absolve themselves of the responsibility of addressing environmental problems if they are portrayed as a natural consequence of economic development that have to be accepted, or as less serious in comparison with other places. We therefore assert the need for more detailed studies of vernacular understandings of climate change that draw attention to the sociopolitics of everyday communicative practices. Neglect of the situated, cultural and politically charged nature of climate change will likely only lead to policy that does not resonate with citizens in ways that motivate action, if related concerns are not addressed.

## Electronic supplementary material

ESM 1 (DOCX 16 kb)

ESM 2 (DOCX 17.5 kb)
