# Supplementary material for: Local climate change cultures: climate-relevant discursive practices in three emerging economies
Source: Clim Change. 2019 Jul 9;163(1):63–82. doi: 10.1007/s10584-019-02477-8 (PMC7704444; doi:10.1007/s10584-019-02477-8)
Supplement: Supplementary file 1 — (DOCX 16 kb) [file 10584_2019_2477_MOESM1_ESM.docx]

**Supplementary Material. Semi-structured interview protocol**

The following protocol was used as a basis for interviews in all three countries. The extracts presented in the paper were taken almost exclusively from responses to Section 2; principally question 2a, which was designed to elicit perceptions of environmentally-relevant issues.

**Introduction:**

1. Can you tell me about things you value in your life?
2. What do you enjoy spending your time doing?
3. What is important to you in your life?

**Environmental perspectives:**

1. Can you tell me what the environment means to you personally?
2. How important are environmental issues/values to you in the context of your life?
3. Can you recall any environmental experiences from your life?
   1. Have you heard of climate change or global warming?

**Characteristics of environmental behavior:**

1. Can you name me as many environmentally-friendly behaviors that you can think of?
2. Can you tell me about the kinds of environmentally-friendly things have you done?
3. Can you tell me about why you do those things?
4. How often do you do these things?
5. Where do you do these things?
6. How does performing these behaviors make you feel?
7. When did you start doing each of these things?
8. What made you start doing each of them?
9. Do you feel that you have changed as a person since starting to perform these behaviors?
10. When you think about all the pro-environmental behaviors that you do in your life, do you see them as similar or different?

**Recollections of spillover:**

1. a. Can you tell me whether doing these behaviors has affected other areas of your life?
2. Can you remember whether in the past doing an environmentally-friendly behavior has caused you to do another environmentally-friendly behavior?

**Perceptions of pro-environmental behavioral norms:**

1. a. How would you say you compare to other people you know in terms of the environmentally- friendly behaviors you do?
2. What do you think about people who do not do things that are environmentally-friendly?
3. What do you think about people who do things that are environmentally-friendly?
4. Would you like to be more like these people in your own life?
5. What kinds of things prevent you from becoming more like them?
