# Supplementary material for: Local climate change cultures: climate-relevant discursive practices in three emerging economies
Source: Clim Change. 2019 Jul 9;163(1):63–82. doi: 10.1007/s10584-019-02477-8 (PMC7704444; doi:10.1007/s10584-019-02477-8)
Supplement: Supplementary file 2 — (DOCX 17.5 kb) [file 10584_2019_2477_MOESM2_ESM.docx]

Supplementary Material

Main structured interview questions

1. Can you tell me about things you value in your life? What do you enjoy spending your time doing? What is important to you?
2. Can you tell me what the environment means to you?
3. How important are environmental issues/values to you in the context of your life?
4. What is the environment like around here?
5. Are you concerned about the environment?
6. Is there anything you can do personally to address those concerns?
7. Do you think that the environment will change in the future?
8. Have you heard of climate change? If so, what have you heard?
9. Where do you get your information about climate change?
10. Do you feel that there is anything that you personally can do about climate change?
